# Specific Premature Groups Have Better Benefits When Treating Apnea With Caffeine Than Aminophylline/Theophylline

**DOI:** 10.3389/fped.2022.817624

**Published:** 2022-02-24

**Authors:** Yi-Chieh Lin, Yin-Ling Tan, Ting-An Yen, Chien-Yi Chen, Po-Nien Tsao, Hung-Chieh Chou

**Affiliations:** ^1^Department of Pediatrics, National Taiwan University Children Hospital, National Taiwan University College of Medicine, Taipei, Taiwan; ^2^Department of Pediatrics, Taoyuan General Hospital, Ministry of Health and Welfare, Taoyuan, Taiwan

**Keywords:** caffeine, aminophylline/theophylline, apnea of prematurity, efficacy, side effects

## Abstract

**Background:**

Methylxanthines (caffeine; aminophylline/theophylline) are commonly used for apnea of prematurity (AOP) treatment. We aimed to compare the efficacy and adverse effects of caffeine and aminophylline/theophylline.

**Methods:**

A retrospective case–control gestational age-matched study investigates patients born between January 2017 and December 2018, 23–35 weeks gestation with birth weights >500 g treating AOP with caffeine or aminophylline/theophylline.

**Results:**

There were 144 cases (48 in caffeine group and 96 in aminophylline/theophylline group). The median treatment durations were 11 and 17 days in caffeine and aminophylline/theophyllinegroup (*p* = 0.002). When tachycardia is defined as heart rate ≥160 bpm, the rates were 8.3 and 34.4% in caffeine and control group (*p* = 0.001). When tachycardia is defined as 10 bpm over baseline heart rate, the rates were 41.7 and 63.5% in caffeine and aminophylline/theophylline group (*p* = 0.01). Stratified by gestational age and sex, significant reductions in tachycardia rates with caffeine than with theophylline were limited to male infants and infants born at <30 weeks gestation.

**Conclusions:**

For apnea treatment, caffeine has greater efficacy and fewer tachycardia than aminophylline/theophylline, especially in male infants and infants born at <30 weeks gestation.

## Introduction

Premature infants have structurally and functionally immature organs. An immature respiratory control system is less responsive to changes in carbon dioxide levels, contributing to apnea (1). This is one of the most common phenomena impacting premature infants in the neonatal intensive care unit (NICU) (2). Apnea of prematurity (AOP) is defined as a cessation of breathing for ≥20 or <20 s accompanied by bradycardia (heart rate <100 bpm) or desaturation (3–6), which could be classified into central, obstructive, and mixed type. Among them, mixed type is the most common in premature infants (6, 7). Repeated significant hypoxia/hypoxemia episodes may be increased the risk for retinopathy of prematurity (ROP) (8, 9) and future neurodevelopmental impairment (5, 7, 8, 10, 11).

Therapeutic methods for AOP management include non-medication and medication therapies. Non-medication treatments, such as prone positioning, continuous positive airway pressure, and intermittent positive airway pressure ventilation, are more effective for obstructive-type apnea; however, evidence of the effects of these interventions for central-type apnea is limited (5, 6, 12). Additionally, positive pressure ventilation may increase the risk of ventilator-induced lung injury (13). Alternatively, medications are an effective choice for AOP treatment.

The most common pharmacologic treatment of AOP is methylxanthines, which have been used for decades (5, 14–16). Methylxanthines, including caffeine and aminophylline/theophylline, work as adenosine receptor antagonists. Although the detailed mechanism is not fully understood (2, 5, 8, 16–19), methylxanthines can stimulate the central drive of the respiratory impulse, increasing diaphragmatic contractility (20). Some adverse effects of methylxanthine were also reported, including tachycardia, feeding intolerance, hypertension, hyperglycemia, and hyponatremia (21).

Many studies have compared the efficacy and incidence of adverse events between caffeine and theophylline in the treatment of AOP. Most of these studies concluded that caffeine has similar efficacy as aminophylline/theophylline in decreasing apnea frequency. However, caffeine has a wider therapeutic range, a longer half-life, and fewer adverse events than aminophylline/theophylline (2, 7, 8, 16, 18, 22). Therefore, caffeine has become the first-choice medication for AOP treatment (23).

Caffeine was introduced in this context in Taiwan in February 2018. Based on the limited practice in our population, we wanted to explore the efficacy and adverse effects of caffeine and theophylline in the context of AOP management. In addition, in past studies, investigators usually enrolled infants less than 34 weeks gestational age (2, 24–28). However, they did not further stratify by age to evaluate which group exhibited greater efficacy and fewer adverse effects. Furthermore, caffeine is a self-paid medication in our country; therefore, we wanted to identify the target group that exhibited better efficacy and fewer adverse effects when treated with caffeine.

This study aimed to compare the therapeutic effects, adverse effects, and morbidities between caffeine and aminophylline/theophylline treatments, with particular emphasis on identifying candidates for whom better efficacy would be achieved and who would have fewer adverse effects with methylxanthine for treating AOP.

## Materials and Methods

In this retrospective, gestational age-matched, case–control study, infants born at 23–35 weeks gestation and admitted to the National Taiwan University Children Hospital NICU at 3 days old or younger between February and December 2018 (caffeine group) and between January 2017 and February 2018 (aminophylline/theophylline group) were enrolled. For every infant who received caffeine, there were two gestational age-matched infants who received aminophylline/theophylline (historical reference group).

Infants with clinically significant AOP and gestational ages between 23 and 35 weeks with birthweights >500 g were included. Neonates who had secondary apnea (29), died before discharge, had apnea combined with significant congenital abnormalities (including congenital heart disease), and those who were treated with both caffeine and aminophylline/theophylline during hospitalization were excluded.

The loading dose of caffeine citrate (Peyona, 20 mg/ml solution for infusion and oral administration; Alfasigma, Italy) was 20 mg/kg, followed by a maintenance dosage of 5 mg/kg/dose once per day. The dosage was titrated by 10 mg/kg/dose per day until clinical effectiveness was reached. The loading dose of aminophylline/theophylline (aminophylline, 25 mg/ml; Purzer, theophylline anhydrous, 5.34 mg/ml) was 5 mg/kg, followed by a maintenance dosage of 3 mg/kg/day divided into three doses a day. If there were no apnea episodes for seven consecutive days, medication was discontinued.

All clinically relevant characteristics, including sex, gestational age, birth weight, delivery method, single or multiple births, Apgar scores (1 and 5 min), completeness of antenatal steroids, presence of chorioamnionitis, and initiation date of AOP medication, were recorded in both groups.

The primary outcomes were difference in the duration of medication use and incidence of tachycardia between the caffeine and reference groups. To minimize the bias due to wide variability in heart rate among infants, tachycardia was defined according to the following criteria: heart rate >160 bpm, heart rate >10 bpm over individual baseline heart rate, and heart rate >20% of baseline heart rate for 8 h consecutively in the first 7 days of medication use. The baseline heart rate was defined as the average heart rate of 8 h before methylxanthine initiation; the heart rate was recorded every hour. For infants who had AOP within 8 h after birth, the baseline heart rate was the average heart rate before medication initiation. Recording of the heart rates continued during procedures. The device we used for record heart rates and SpO_2_ was Philips IntelliVue MX 800.

The secondary outcomes were durations of intubation, and noninvasive ventilation [including nasal intermittent positive pressure ventilation (NIPPV), nasal continuous positive airway pressure (NCPAP)], total ventilator days during hospitalization, incidence of bronchopulmonary dysplasia (BPD), any grade intraventricular hemorrhage (IVH), post-hemorrhagic hydrocephalus, ventriculomegaly, and ROP requiring treatment. Bronchopulmonary dysplasia was defined according to the definition of National Institutes of Health 2011 workshop as infants who require oxygen supplementation for at least 28 days. Assessment was made at 36 weeks' post-menstrual age or upon discharge (30). The feeding policy in our NICU is a daily increment of 20 ml/kg/day if fair digestive condition is observed in patients. The feeding protocol is suspended or slowed down once abdominal fullness or residual volume more than half feeding amount is noticed. The risk of feeding intolerance increased with prolonged time to full feeding. Therefore, in this study, time to full feeding was utilized as an indicator of feeding intolerance.

### Statistical Analysis

All normally distributed categorical variables, such as sex, delivery mode, single or multiple delivery, and incidence of BPD, IVH, ROP treated with intravitreal injection of anti-vascular endothelial growth factor agents, and tachycardia, were analyzed using a chi-squared test. All normally distributed continuous variables, including gestational age, birth weight, and 1- and 5-min Apgar scores, are presented as the mean ± standard deviation and were analyzed using an independent *t*-test. Non-normally distributed data, including duration of medication use, intubation days, non-invasive ventilation days, total ventilation days, and time to full enteral feeding, were expressed as the median and interquartile range and analyzed using a Mann–Whitney U test. Fisher's exact test was applied to analyze parameters when the number of cases was <5, such as chorioamnionitis, post-hemorrhagic hydrocephalus, and ventriculomegaly. All analyses were performed in SPSS, version 19 (IBM Corp. Released 2010, IBM SPSS Statistics for Windows, Version 19.0. Armonk, NY). *P* <0.05 was considered to indicate statistical significance. The required sample size was 34 when power is 0.8, the alpha error is 0.05, and the effect is 0.5, as calculated by G^*^Power 3.1.9.7.

## Results

**Figure 1:**
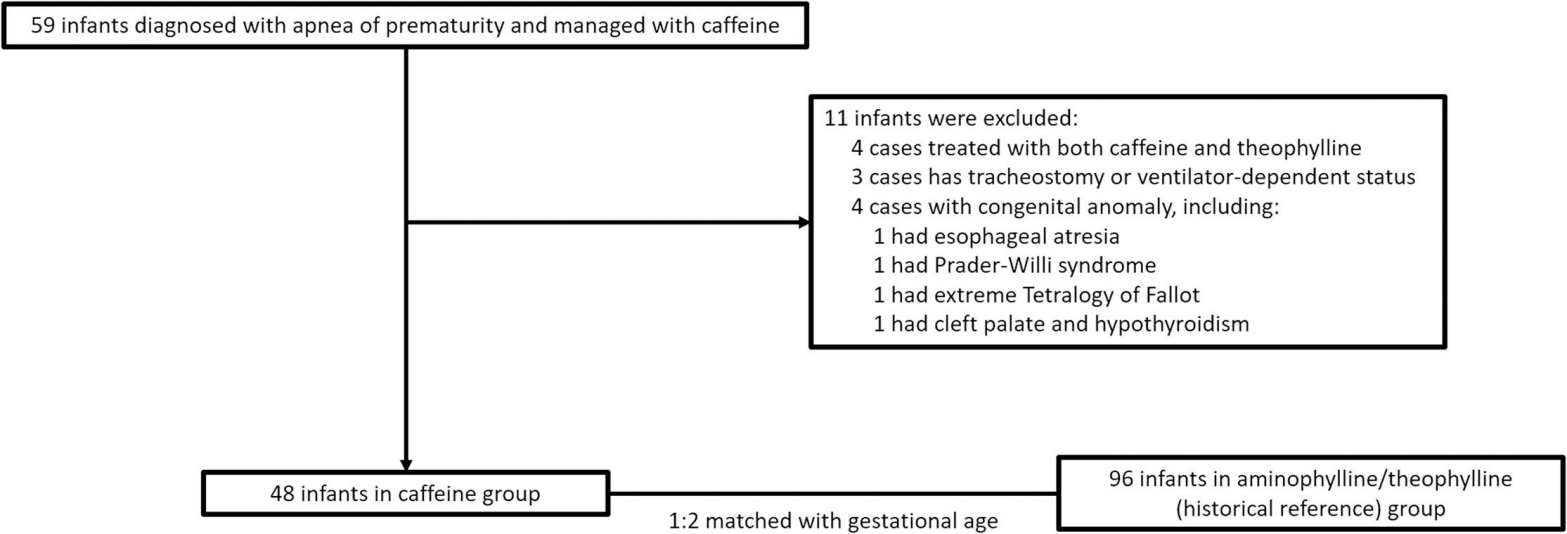
Schematic of patients selection process.

A total of 59 infants with AOP treated with caffeine were examined. We excluded four infants who had received both caffeine and aminophylline/theophylline during hospitalization, four infants with significant congenital abnormalities (one with esophageal atresia, one with Prader–Willi syndrome, one with extreme tetralogy of Fallot, and one with cleft palate and lip and hypothyroidism), and three infants who were discharged with home ventilators [two were treated with bilevel positive airway pressure (BiPAP), one underwent tracheostomy with BiPAP, and one was treated with a high-flow ventilator]. Finally, 48 patients were enrolled in the caffeine group. Ninety-six gestational age-matched patients treated with aminophylline/theophylline were enrolled in the control group (Figure 1).

When the eligible population was defined, the reference population was matched for gestational age in a 1:2 ratio and enrolled.

No differences in gestational age or birth weight were observed between the caffeine and reference groups (*p* = 0.79 and *p* = 0.62). Other baseline demographics and clinical characteristics, such as sex, incidence of chorioamnionitis, and time of treatment initiation, were all comparable between the caffeine and referencegroups (Table 1).

**Table 1:** Baseline demographics and clinical characteristics of the patients.

	**Caffeine**	**Reference group^***d***^**	***P***
	**(*N* = 48)**	**(*N* = 96)**	
Sex			0.55^*b*^
Male	28 (58.3%)	51 (53.1%)	
GA	30.08 ± 2.73	29.96 ± 2.57	0.79^*a*^
BBW	1293.1 ± 392.0	1329.9 ± 453.9	0.62^*a*^
Delivery, NSD	7 (14.6%)	26 (27.1%)	0.09^*b*^
Birth, single	32 (66.7%)	69 (71.9%)	0.52^*b*^
Chorioamnionitis	5 (10.4%)	6 (6.3%)	0.51^*c*^
Apgar score (1 min)	5 (4–7)	6 (4–7)	0.89^*a*^
Apgar score (5 min)	8 (7–9)	8 (7–9)	0.92^*a*^
When to start use (day)	4.25 ± 4.47	3.05 ± 2.75	0.09^*a*^

*BBW, birth body weight; GA, gestational age; NSD, normal spontaneous delivery*.

a *t-test*;

b *chi-squared test*;

c *Fisher's exact test*;

d *aminophylline/theophylline*.

For primary outcomes, the median treatment durations were 11 and 17 days in the caffeine and control groups, respectively (*p* = 0.002). Tachycardia rates according to the definition of heart rate ≥160 bpm were 8.3 and 34.4% in the caffeine and referencegroups, respectively (*p* = 0.001; Table 2). When tachycardia was defined as heart rate >10 bpm over baseline, the rates were 41.7 and 63.5% in the caffeine and referencegroups, respectively (*p* = 0.01; Table 2).

**Table 2:** Comparison of efficacy, morbidities, and adverse effects.

	**Caffeine**	**Reference group^***d***^**	***p***
	**(*N* = 48)**	**(*N* = 96)**	
Primary outcomes			
Medication duration (day)	11 (8–19)	17 (10–37)	**0.002** ^*a*^
Tachycardia, HR > 160 bpm	4 (8.3%)	33 (34.4%)	**0.001** ^*b*^
Tachycardia, baseline HR + 10 bpm	20 (41.7%)	61 (63.5%)	**0.01** ^*b*^
Tachycardia, >20% baseline HR	3 (6.3%)	11 (11.5%)	0.39^*b*^
Secondary outcomes			
Intubation (day)	0 (0–0)	0 (0–0)	0.36^*a*^
Noninvasive ventilation (day)	30 (4–70)	14 (4–44)	0.28^*a*^
Total ventilation days	31 (4–73)	14 (4–44)	0.29^*a*^
BPD	12 (25%)	13 (13.5%)	0.09^*b*^
IVH	4 (8.3%)	19 (19.8%)	0.09^*b*^
PHH	0 (0.00%)	1 (1.00%)	1.00^*c*^
Ventriculomegaly	2 (4.2%)	5 (5.2%)	1.00^*c*^
ROP-IVIA	4 (8.3%)	4 (4.2%)	0.44^*b*^
Time to full enteral feeding	9 (7–13)	10 (7–15)	0.47^*a*^

*BPD, bronchopulmonary dysplasia; HR, heart rate; IVH, intraventricular hemorrhage; IVIA, intravitreal injection of bevacizumab (Avastin); PHH, post-hemorrhagic hydrocephalus; ROP, retina of prematurity*.

a *Mann–Whitney U test*;

b *chi-squared test*;

c *Fisher's exact test*;

d *aminophylline/theophylline. The bold value means that the results have statistic difference*.

For secondary outcomes, short intubation durations were noted in both groups (*p* = 0.36), and the median durations of mechanical and non-invasive ventilation (NCPAP and NIPPV) were 30 and 14 days in the caffeine and reference groups, respectively (*p* = 0.28). The total ventilation days were not significantly different between the two groups (31 vs. 14 days, *p* = 0.29). The median time to full enteral feeding in the caffeine group was 9 days, whereas that in the referencegroup was 10 days (*p* = 0.47). Additionally, the long-term outcomes (BPD, IVH, post-hemorrhagic hydrocephalus, ventriculomegaly, and severe ROP requiring intravitreal injection of Avastin) showed no significant differences between the two groups (Table 2).

After primary analysis, we stratified the study population by gestational age and sex into subgroups to compare the effectiveness for AOP treatment and the incidence of tachycardia between the caffeine and referencegroups. We classified patients by gestational age from 23 to 34 weeks. Among patients with gestational age ≤30 weeks, caffeine demonstrated a shorter medication duration than aminophylline/theophylline in infants with a younger gestational age (14.5 days vs. 28.5, *p* = 0.004, Table 3), whereas no significant differences were observed in infants with an older gestational age (9 days vs. 10, *p* = 0.07). The tachycardia rates in the caffeine group were significantly lower than those in aminophylline/theophylline group in infants with a younger gestational age (15.4 vs. 51.8%, *p* = 0.002 in HR > 160/bpm; 38.5 vs. 67.9%, *p* = 0.012 in HR > baseline + 10/bpm, Table 3). In infants with an older gestation age, the incidence of tachycardia did not statistically differ according to the use of caffeine or aminophylline/theophylline (0 vs. 6.5%, *p* = 0.29 in HR > 160/bpm; 16.1 vs. 37.1%, *p* = 0.36 in HR > baseline + 10/bpm, Table 3). We also analyzed if ventilator use in gestation age subgroups had statistic significant difference. There was no statistic difference was noted (Table 4). Additionally, when stratified by sex, caffeine was more beneficial than aminophylline/theophylline in males (11 vs. 20 days, *p* = 0.000, Table 5). This phenomenon was not noted in females (12 vs. 15 days, *p* = 0.50, Table 5). Regarding adverse effects, premature males had significantly fewer tachycardia episodes after receiving caffeine than those receiving aminophylline/theophylline (3.6 vs. 39.2%, *p* = 0.000, Table 5), but this effect was not observed in females (42.9 vs. 72.5%, *p* = 0.015, Table 5).

**Table 3:** Target group stratified by gestational age.

	**GA** **≤30 weeks**			**GA** **>** **30 weeks**		
	**Caffeine**	**Reference group^***c***^**	***p***	**Caffeine**	**Reference group^***c***^**	***p***
	***N* = 26**	***N* = 56**		***N* = 22**	***N* = 40**	
Medication duration (day)	14.5 (10–32)	28.5 (14–46)	**0.004** ^***a***^	9 (7–11)	10 (8–15)	0.07^*a*^
Tachycardia						
HR > 160 bpm	4 (15.4%)	29 (51.8%)	**0.002** ^***b***^	0 (0.0%)	4 (6.5%)	0.29^*b*^
>Baseline HR + 10 bpm	10 (38.5%)	38 (67.9%)	**0.012** ^***b***^	10 (16.1%)	23 (37.1%)	0.36^*b*^

*HR, heart rate*.

a *Mann–Whitney U test*;

b *chi-squared test*;

c *aminophylline/theophylline. The bold value means that the results have statistic difference*.

**Table 4:** Ventilation using stratified by gestational age.

	**GA** **≤30 weeks**			**GA** **>** **30 weeks**		
	**Caffeine**	**Reference group^***b***^**	***p***	**Caffeine**	**Reference group^***b***^**	***p***
	***N* = 26**	***N* = 56**		***N* = 22**	***N* = 40**	
Intubation (day)	0 (0–6)	0 (0–0)	**0.074** ^*a*^	0 (0–0)	0 (0–0)	**0.128** ^*a*^
Noninvasive ventilation (day)	62 (34–82)	42 (16–76)	**0.99** ^*a*^	4 (2–14)	4 (2–7)	**0.472** ^*a*^
Total ventilation days	69 (35–85)	42 (16–77)	**0.079** ^*a*^	4 (2–14)	4 (2–7)	**0.573** ^*a*^

a *Mann–Whitney U test*;

b *aminophylline/theophylline. The bold value means that the results have statistic difference*.

**Table 5:** Target group stratified by sex.

	**Male**			**Female**		
	**Caffeine**	**Reference group^***c***^**	***p***	**Caffeine**	**Reference group^***c***^**	***p***
	***N* = 28**	***N* = 51**		***N* = 20**	***N* = 45**	
Medication duration (day)	11 (8–14)	20 (11–41)	**0.000** ^***a***^	12 (8–30)	15 (9–25)	0.503^*a*^
Tachycardia						
HR > 160 bpm	1 (3.6%)	20 (39.2%)	**0.000** ^***b***^	3 (15.0%)	13 (28.9%)	0.351^*b*^
>Baseline HR + 10 bpm	12 (42.9%)	37 (72.5%)	**0.015** ^***b***^	8 (40.0%)	24 (53.3%)	0.422^*b*^

*HR, heart rate*.

a *Mann–Whitney U test*;

b *chi-squared test*;

c *aminophylline/theophylline. The bold value means that the results have statistic difference*.

## Discussion

Our investigation demonstrated that caffeine was more effective in treating AOP and resulted in fewer tachycardia episodes than aminophylline/theophylline, especially in male infants and infants born at <30 weeks gestation. To the best of our knowledge, our study is one of the few investigations that clarified the target groups where caffeine may be more effective while having a lower risk of developing adverse effects than aminophylline/theophylline.

The benefits of using methylxanthines for AOP management have been well-documented (1, 7, 14, 20, 31, 32). Studies have compared the efficacy and adverse effects between aminophylline/theophylline and caffeine. The representative parameters chosen to indicate the efficacy of these medications are important to consider before reaching definitive conclusions. In some studies, infants were followed for 7 days, and the frequency of daily apnea episodes was assessed after medication initiation. These studies demonstrated no significant difference between caffeine and aminophylline/theophylline in decreasing apnea frequency (2, 7, 17, 24–26, 33). However, in our investigation, apnea episode recordings might have been missed due to the retrospective study design. Other studies using oxygen demand and duration to assess the efficacy of medications in such patients are inconclusive (5, 21). Compared to those prospective studies, in the present study, oxygen demand was not considered as a parameter to evaluate the efficacy of both medications because of strict ventilation and oxygen utilization policies in our NICU setting. Instead of the mean reduction in apnea episodes and oxygen demand, treatment duration was applied to assess efficacy of caffeine and aminophylline/theophylline. The results showed that using caffeine to treat AOP resulted in a shorter medication duration than using aminophylline/theophylline (11 vs. 17 days, *p* = 0.002), indicating that caffeine had more efficacy than theophylline. This results also imply that caffeine decreased the number of apnea episodes earlier than theophylline. This finding was different from previous results (2, 7, 17, 24–26, 33).

The duration of ventilator use, especially NCPAP, had controversial results in previous investigations (2, 5, 17). In our study, there were no significant differences between the caffeine and aminophylline/theophylline groups in the duration of ventilation use. In the existing literature where the incidences of BPD, patent ductus arteriosus ligation, necrotizing enterocolitis, IVH, post-hemorrhagic hydrocephalus, periventricular leukomalacia, and treated ROP were considered as long-term outcomes of caffeine and aminophylline/theophylline treatment, there were no significant differences between the two groups in the existing literature (2, 5, 17, 18, 21, 24, 27, 28, 34), which is comparable with our findings (Table 2).

There is no standard definition of tachycardia in the literature. Some studies defined tachycardia as a heart rate > 180 bpm (5, 33), whereas others defined tachycardia as a heart rate > 200 bpm (28). Most studies recorded daily average heart rate within 7–14 days after medication administration. The majority of past study results showed that the tachycardia incidence was higher in the aminophylline/theophylline group than in the caffeine group (2, 24, 25, 27). However, the use of absolute heart rate and relative heart rate changes to defined tachycardia is more precise and less affected by extreme values. Thus, we applied three different definitions of tachycardia simultaneously to minimize the bias of different individual baseline heart rates. With those three definitions of tachycardia, a similar finding was derived: a higher incidence of tachycardia was identified in the aminophylline/theophylline group than in the caffeine group (8.3 vs. 34.4%, *p* = 0.001 in HR > 160 bpm, 41.7 vs. 63.5%, *p* = 0.01 in HR > baseline HR + 10 bpm).

One of the major findings in our investigation is that in the context of caffeine use, the medication duration for AOP management was significantly shorter and the number of tachycardia episodes was remarkably lower than with the use of aminophylline/theophylline in patients with AOP whose gestational age was <30 weeks. However, these findings did not appear in neonates with a gestational age >30 weeks. It is clear that the rate of methylxanthine metabolism increases with age (21, 35). However, most previous studies enrolled infants with gestational ages <35 weeks (2, 26–28). There was no clear cutoff point of the gestational age of newborns who were more responsive to caffeine than aminophylline/theophylline for AOP management. Our results demonstrated important evidence that for AOP management, caffeine was a better medication choice than aminophylline/theophylline for infants with younger gestational ages.

Furthermore, the other major finding in our study was that similar effects of caffeine and aminophylline/theophylline, including shorter medication duration and fewer tachycardia episodes, were demonstrated in male infants. The same effect was not shown in female infants. Al-Alaiyan et al. aimed to discover whether the rate of caffeine metabolism during premature stages was influenced by postnatal age, birth weight, study weight, gestational age, post-conceptual age, and sex (35). The authors analyzed the caffeine metabolic pathway and found that caffeine metabolism in premature infants increased with postnatal age, and female infants had a higher rate of caffeine metabolism than male infants. Our findings that caffeine had greater efficacy in male infants and resulted in less frequent tachycardia episodes may be explained by different metabolic rates between the sexes and gestational ages.

Our study had some limitations. First, its retrospective design might contribute to potential bias because of missing medical records. The two medication groups were enrolled at different periods. However, subjects were chosen in similarly recent years to minimize the biases caused by different medical care policies during earlier periods. Second, data in our hospital is preserved for a limited period in electronic records, which made it difficult to match the gestational age perfectly. When patients were stratified by gestational age of 30 weeks, one caffeine case may not have been matched with two aminophylline/theophylline cases. However, the ratio of subjects in the caffeine and aminophylline/theophylline groups was nearly 1:2. Third, our patients were matched by gestational age, yet they could not be matched by body weight due to the retrospective design. Fourth, even the case number is larger than previous studies, it is still a small case number then. Fifth, stratification according to gestational age and sex was not pre-specified. Other factors that may be considered as limitations include that adverse effects such as hyperglycemia and hyponatremia mentioned in previous studies (5, 27, 34, 36) were not analyzed in this investigation because routine blood tests were not obtained after the patients' clinical condition stabilized. Furthermore, methylxanthine is a known central nervous system stimulant, and it may be related to seizure or jitteriness (7). None of our participants had neurological presentations such as jitteriness or seizure. Finally, the rate of caffeine metabolism could not be monitored according to different gestational age or sex due to the unavailability of serum levels of caffeine in our medical service.

Although many prospective randomized controlled trials have been performed, it is rare that studies aim to identify target groups that respond to methylxanthine; thus, more studies are required for better comparisons involving methylxanthine in Taiwan.

In conclusion, our study had same view with previous study that using caffeine in treating AOP had less tachycardia episodes than aminophylline/theophylline (2, 24, 25, 27). However caffeine is more effective and reduced the number of apnea episodes earlier in our study, which is different from other studies (2, 7, 17, 24–26, 33). Further, compared to previous studies, our investigation finds there are specific sub-populations of premature infants, male infants and infants with a gestational age ≤ 30 weeks, who might have the greatest benefit from using caffeine in treating AOP instead of aminophylline/theophylline.

Our results may suggest that for these two infant populations, caffeine is more effective and has fewer adverse effects in the treatment of AOP.

## Data Availability Statement

The raw data supporting the conclusions of this article will be made available by the authors, without undue reservation.

## Ethics Statement

The studies involving human participants were reviewed and approved by the Institutional Review Board of National Taiwan University Hospital. Written informed consent from the participants' legal guardian/next of kin was not required to participate in this study in accordance with the national legislation and the institutional requirements.

## Author Contributions

Y-CL, Y-LT, T-AY, C-YC, P-NT, and H-CC: conceptualization. Y-CL and Y-LT: data curation and investigation. Y-CL, Y-LT, P-NT, and H-CC: formal analysis. Y-CL, C-YC, P-NT, and H-CC: methodology. T-AY, C-YC, P-NT, and H-CC: project administration. Y-CL and C-YC: resources. Y-CL, Y-LT, and C-YC: software. T-AY, P-NT, and H-CC: supervision. Y-CL and H-CC: validation. Y-CL and T-AY: visualization. Y-CL: writing—original draft preparation. P-NT and H-CC: writing—review and editing. All authors contributed to the article and approved the submitted version.

## Conflict of Interest

The authors declare that the research was conducted in the absence of any commercial or financial relationships that could be construed as a potential conflict of interest.

## Publisher's Note

All claims expressed in this article are solely those of the authors and do not necessarily represent those of their affiliated organizations, or those of the publisher, the editors and the reviewers. Any product that may be evaluated in this article, or claim that may be made by its manufacturer, is not guaranteed or endorsed by the publisher.
